# Novel Compound-Heterozygous Variants in PYCR1 Expand the Variant Spectrum of Autosomal Recessive Cutis Laxa

**DOI:** 10.3390/genes17091129

**Published:** 2026-09-16

**Authors:** Xin Wang, Zhuran Zhao, Weike Cheng, Jing Liu, Shimin Zhang, Yanhui Dong, Shuai Xu

**Affiliations:** 1 Department of Pediatrics, Peking University People’s Hospital, Beijing 100044, China; 2 Epilepsy Center, Peking University People’s Hospital, Beijing 100044, China; 3 National Health Commission Key Laboratory of Reproductive Health, Institute of Child and Adolescent Health, School of Public Health, Peking University, Beijing 100191, China; 4 Department of Spinal Surgery, Peking University People’s Hospital, Beijing 100044, China

**Keywords:** PYCR1, autosomal recessive cutis laxa, compound heterozygosity, functional evidence, variant interpretation, RNA splicing

## Abstract

Background and Objectives: Autosomal recessive cutis laxa (ARCL) is a genetically heterogeneous group of connective-tissue disorders characterized by loose, inelastic skin and variable systemic involvement. Biallelic variants in PYCR1 are associated with PYCR1-related ARCL, including ARCL type IIB (OMIM #612940) and type IIIB (OMIM #614438). We aimed to characterize the clinical and molecular findings in one affected family and provide segregation and variant-specific functional evidence for two previously unreported PYCR1 variants. Methods: The proband and family underwent whole-exome sequencing with copy-number analysis and Sanger confirmation. The splice-site variant was evaluated by qualitative RT-PCR and Sanger sequencing of endogenous peripheral-blood PYCR1 transcripts, whereas the frameshift variant was studied in a heterologous expression system by qPCR and Western blotting. Neuroimaging, exploratory EEG spectral analysis, and public developmental transcriptomic/single-cell resources were used for complementary phenotypic context. Results: The 16-year-old proband had thin lax skin with prominent superficial veins, generalized joint hypermobility, facial dysmorphism, reduced muscle bulk, and motor developmental delay. Chinese WAIS-IV showed a markedly uneven profile (VCI 52; PRI 94; FSIQ 75). Two previously unreported PYCR1 variants were identified in trans: maternally inherited NM_006907.4:c.139-1G>A and paternally inherited NM_006907.4:c.724_725del, p.(Leu242AlafsTer31). Endogenous RNA analysis identified exon 3 skipping associated with c.139-1G>A (r.139_318del), predicting p.(Met48_Lys107del). The c.724_725del construct showed consistently reduced steady-state abundance of the truncated PYCR1 protein relative to WT across three independent experiments. Both variants were classified as likely pathogenic using the ACMG/AMP framework with relevant ClinGen refinements. Brain MRI revealed irregular morphology of the anterior corpus callosum. Discussion: These two previously unreported variants extend the PYCR1 variant spectrum and are supported by complementary segregation, RNA-level, and protein-level evidence. The neurodevelopmental observations provide additional phenotypic context and are interpreted descriptively.

## 1. Introduction

Autosomal recessive cutis laxa (ARCL) comprises a genetically heterogeneous group of connective-tissue disorders characterized by loose, inelastic skin and variable systemic involvement [1,2,3,4,5,6,7,8,9,10]. Biallelic variants in PYCR1 are associated with ARCL type IIB (OMIM #612940) and ARCL type IIIB (OMIM #614438). PYCR1 (NM_006907.4; MANE Select transcript ENST00000329875.13), located at 17q25.3, comprises seven exons separated by six introns and encodes the 319-amino-acid mitochondrial enzyme pyrroline-5-carboxylate reductase 1 [11,12,13,14,15,16,17,18,19]. PYCR1 catalyzes the NAD(P)H-dependent conversion of pyrroline-5-carboxylate to proline, linking proline biosynthesis with extracellular-matrix homeostasis, mitochondrial redox balance, and cellular stress responses [20,21,22]. Previous studies have suggested possible genotype–phenotype trends in PYCR1-related disease, including greater clinical severity, on average, among variants affecting exons 4–6; however, considerable phenotypic overlap occurs across variant locations and classes [15]. Accordingly, reported genotype–phenotype associations are best interpreted as emerging trends rather than deterministic relationships.

In this study, we investigated a patient with features suggestive of ARCL who carried two previously unreported compound-heterozygous PYCR1 variants. We integrated family segregation, endogenous RNA analysis, expression-construct studies, detailed clinical and neurodevelopmental phenotyping, and complementary public transcriptomic and single-cell resources to refine variant interpretation and place the findings within the reported PYCR1-related disease spectrum.

## 2. Methods

### 2.1. Study Participant and Clinical Evaluation

A 16-year-old boy with intellectual and developmental delay and physical features suggestive of autosomal recessive cutis laxa (ARCL) was enrolled. Detailed medical, developmental, and family histories were obtained, and a pedigree was constructed. Neurologic evaluation included brain MRI, electroencephalography with power spectral density analysis, and cognitive assessment using the Chinese version of the Wechsler Adult Intelligence Scale, Fourth Edition. To exclude other inherited metabolic disorders, urinary organic acids were analyzed by gas chromatography–mass spectrometry, and amino acids and acylcarnitines in dried blood spots were assessed by tandem mass spectrometry.

### 2.2. Genetic Testing

Peripheral blood samples (2 mL) were collected from the proband, his parents, and his maternal half-sister. Genomic DNA was extracted from whole blood. Trio whole-exome sequencing was performed using NanoWES (Berry Genomics, Beijing, China) target capture followed by 150-bp paired-end sequencing on the DNBSEQ-T7 platform (MGI Tech Co., Ltd., Shenzhen, China). Raw data were processed using CASAVA v1.82; reads were aligned to hg38/GRCh38 using BWA, PCR duplicates were removed using Picard, and SNVs/indels were called using the Verita Trekker^®^ Variants Detection System (Berry Genomics, Beijing, China) together with GATK. Variants were annotated using ANNOVAR and the Enliven^®^ Variants Annotation Interpretation System (Berry Genomics), and exome-derived CNVs were assessed as part of the WES analysis. At least 98.97% of target bases were covered at ≥20×. Population-frequency assessment incorporated gnomAD (https://gnomad.broadinstitute.org/; accessed 8 September 2026), ExAC (via the gnomAD resource; accessed 8 September 2026), the 1000 Genomes Project Phase 3 (https://www.internationalgenome.org/), and the Berry Genomics Chinese population database used by the diagnostic laboratory. Disease resources included ClinVar (https://www.ncbi.nlm.nih.gov/clinvar/; accessed 8 September 2026), HGMD, and OMIM (https://omim.org/; accessed 8 September 2026). Candidate variants were confirmed and familial segregation was assessed by Sanger sequencing. Variants were classified according to the ACMG/AMP framework (Richards et al., 2015), with relevant ClinGen criterion-specific recommendations for loss-of-function, splicing, population-frequency, in trans, and functional evidence [23,24,25,26]. Current guidance was additionally referenced through the ClinGen Variant Classification Guidance resource (https://www.clinicalgenome.org/tools/clingen-variant-classification-guidance/; accessed 8 September 2026). The genomic PCR and cycle-sequencing primers are listed in Table 1.

### 2.3. Endogenous RNA Analysis of the PYCR1 c.139-1G>A Splice Variant

Peripheral-blood RNA from the proband and an unrelated healthy control was analyzed to evaluate the effect of c.139-1G>A on endogenous PYCR1 splicing. Total RNA was extracted from whole blood using a blood total RNA extraction kit (R2112-50; Wuhan Junuode Biotechnology Co., Ltd., Wuhan, China). Residual genomic DNA was removed, and reverse transcription was performed using PrimeScript FAST RT reagent Kit with gDNA Eraser (RR092A; TaKaRa Bio Inc., Kusatsu, Shiga, Japan). Nested RT-PCR was performed using cDNA and PrimerSTAR MAX DNA Polymerase (R045A; TaKaRa). The nested primers annealed within exons 2 and 4 of NM_006907.4, generating an expected 422-bp normally spliced product containing the complete 180-bp exon 3 and portions of the flanking exons; exon 3 skipping therefore generated a 242-bp product. PCR products were resolved by 2% agarose gel electrophoresis. The shorter proband product was excised, purified, subjected to TA cloning, and Sanger sequenced. Sequence alignment to NM_006907.4 was used to identify the splice junction. This endpoint nested RT-PCR assay was used qualitatively for splice-product identification rather than quantitative estimation of transcript proportions. Primer sequences are listed in Table 1.

### 2.4. Expression-Construct Analysis of PYCR1 c.724_725del, p.(Leu242AlafsTer31)

#### 2.4.1. Wild-Type and c.724_725del Expression-Vector Construction

Wild-type (WT) and c.724_725del PYCR1 expression constructs were generated using the pCMV-3XFlag-Neo(EGFP) expression vector. The WT PYCR1 coding sequence was amplified from a synthetic PYCR1 CDS template, and the c.724_725del variant sequence was generated by overlap PCR. WT and variant products were digested with NotI and EcoRI and cloned into the corresponding sites of pCMV-3XFlag-Neo(EGFP), generating N-terminal 3×FLAG-tagged PYCR1 expression constructs. EGFP is expressed independently from the same plasmid and is not fused to PYCR1. Recombinant plasmids were transformed into DH5α competent cells, and positive clones were confirmed by Sanger sequencing. The same WT and c.724_725del constructs were used for qPCR and Western blot analyses. Primer sequences are listed in Table 1.

#### 2.4.2. Cell Culture and Transfection

HEK293T cells were cultured in DMEM (cat. no. 11965092; Gibco, Thermo Fisher Scientific, Waltham, MA, USA) supplemented with 10% fetal bovine serum (cat. no. 10270-106; Gibco) at 37 °C in 5% CO2. Cells were seeded into 6-well plates 1 day before transfection and transfected at approximately 70–90% confluence. For each well, 2.5 micrograms of WT or c.724_725del plasmid DNA was diluted in Opti-MEM (cat. no. 31985-070; Gibco) and mixed with Hieff Trans Universal Transfection Reagent (cat. no. 40802ES30; Yeasen Biotechnology (Shanghai) Co., Ltd., Shanghai, China) according to the manufacturer’s protocol. EGFP fluorescence was used to monitor transfection (Figure S1). Cells were collected 48 h after transfection for qPCR and Western blot analyses.

#### 2.4.3. qPCR

Total RNA was extracted 48 h after transfection using TRIzol (cat. no. 9109; TaKaRa), and cDNA was synthesized using PrimeScript FAST RT reagent Kit with gDNA Eraser (RR092A; TaKaRa). qPCR was performed using the PYCR1 and ACTB primer pairs listed in Table 1. Three independent experiments were performed. Relative construct-derived PYCR1 transcript abundance was calculated using the 2^−ΔΔCq^ method. All experimental observations were retained; no outlier exclusion was applied. Individual values are displayed and summarized descriptively using the mean and observed range.

#### 2.4.4. Western Blotting

Cells were lysed in RIPA lysis buffer (cat. no. P0013B; Beyotime Biotechnology, Shanghai, China), and protein concentrations were measured using a BCA Protein Quantification Kit (cat. no. 20201ES76; YEASEN). Equal amounts of protein were separated by SDS-PAGE and transferred to PVDF membranes (cat. no. 1620177; Bio-Rad Laboratories, Hercules, CA, USA). Membranes were incubated with antibodies against FLAG (cat. no. 2064, 1:2000; Dia-An Biotechnology, Wuhan, China), GFP (cat. no. 2057, 1:1000; Dia-An Biotechnology), and β-actin (cat. no. AC026, 1:2000; ABclonal Technology, Wuhan, China), followed by horseradish peroxidase-conjugated secondary antibodies (Proteintech Group, Inc., Rosemont, IL, USA; 1:10,000). Signals were visualized using a QuickChemi 5100 chemiluminescence imaging system (Monad Biotech Co., Ltd., Wuhan, China). FLAG-PYCR1 band intensities were quantified using ImageJ and normalized to the corresponding β-actin loading control. All three independent experiments were retained, with no observations excluded; densitometry is summarized descriptively using the mean and observed range.

### 2.5. Spatiotemporal Expression Analysis of PYCR1

Adult non-disease tissue expression of PYCR1 was examined using processed data from the GTEx Portal (https://gtexportal.org/; accessed 8 September 2026). Developmental organ-expression profiles were obtained from the Evo-devo mammalian organs resource based on the human RNA-seq dataset of Cardoso-Moreira et al. (ArrayExpress E-MTAB-6814) [27]. Cerebral-organoid scRNA-seq, scATAC-seq, developmental-trajectory, and adult-brain snRNA-seq data were interrogated through ScApeX using the Kanton et al. datasets (E-MTAB-7552, E-MTAB-8089, and E-MTAB-8230) [28]. Expression in 1- and 5-month single-neural-rosette-derived cortical organoids was examined through the Human Organoid Single-Cell Browser using the Wang et al. dataset (GEO GSE118697) [29]. The source dataset contains four 1-month and six 5-month samples; the original study reported 9577 cells after quality control at 1 month and 22,486 cells across the six 5-month samples. Processed expression values, cell-type annotations, and trajectory/pseudotime information were adopted from the corresponding public resources and original studies rather than re-derived from raw sequencing reads in the present study. Dataset accession numbers, biological source, developmental stage, and source-study processing frameworks are specified here and in the corresponding figure legend.

### 2.6. Standard Protocol Approval, Registration, and Patient Consent

This study was approved by the Ethics Committee of Peking University People’s Hospital (approval No. 2024PHB100-001) and conducted in accordance with the Declaration of Helsinki. Written informed consent for research participation, genetic testing, and data analysis was obtained from the legal guardians (parents) of the minor proband. Written informed consent to disclose clinical details, phenotypic information, and all identifiable patient-related data for publication in this journal was also obtained from the patient’s legally authorized representatives. No experiments involving live vertebrates or higher invertebrates were performed. This study is not a clinical trial; therefore, clinical trial registration and protocol registration were not applicable.

### 2.7. Statistical Analysis

For the expression-construct experiments, three independent experiments were performed, and all individual values were retained. Given *n* = 3, qPCR and Western blot densitometry were summarized descriptively using the mean and observed range; no outlier exclusion, formal normality testing, or inferential hypothesis testing was applied.

## 3. Results

### 3.1. Clinical Presentation and Ancillary Investigations

**Figure 1:**
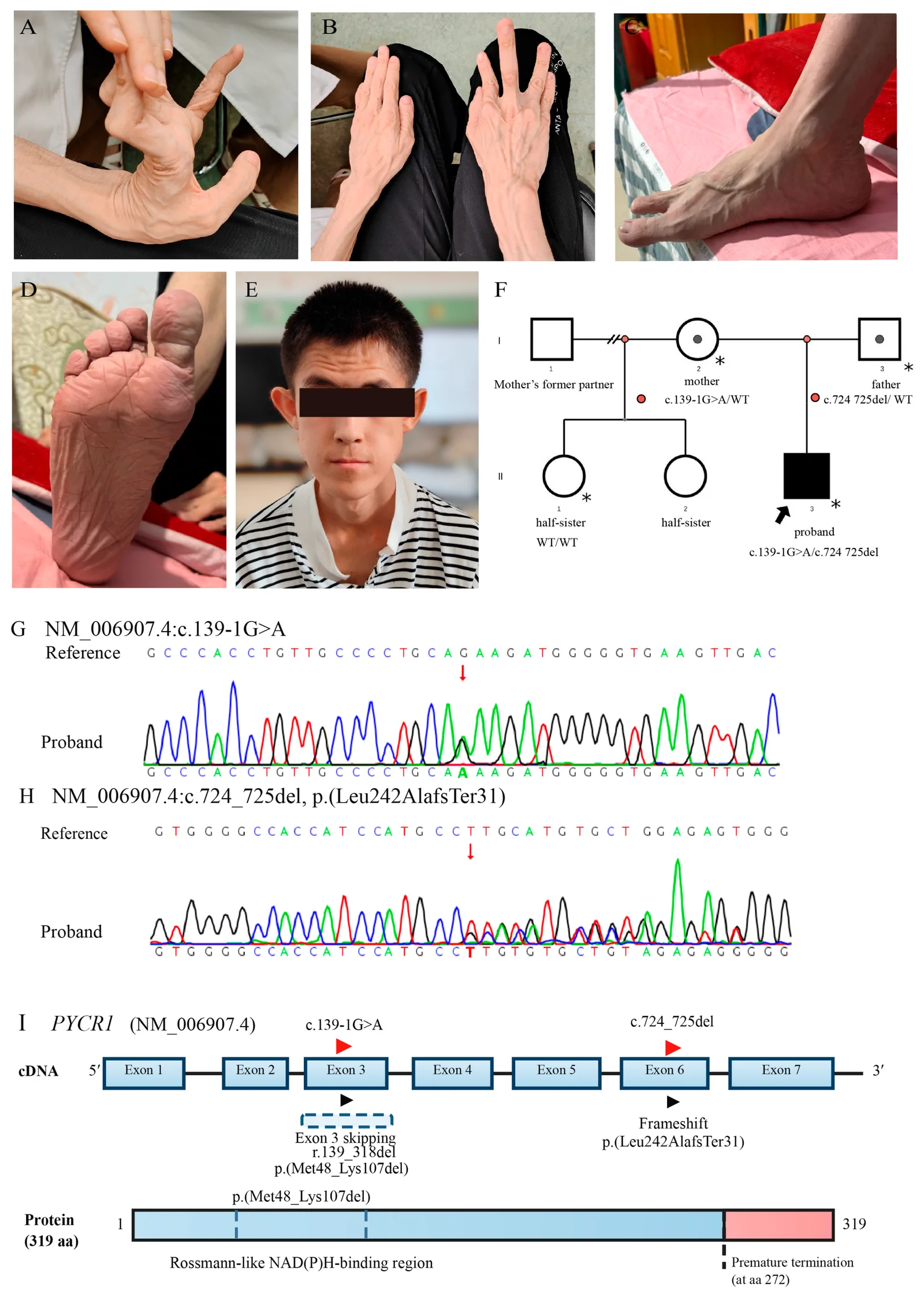
Clinical and molecular findings of the proband with PYCR1-related autosomal recessive cutis laxa. (**A**–**E**) Representative clinical features. (**F**) Pedigree showing the genotypes and segregation of the two PYCR1 variants. c.139-1G>A was maternally inherited, and c.724_725del was paternally inherited, establishing the variants in trans. The maternal half-sister carried neither variant; NT, not tested. (**G**,**H**) Sanger sequencing of the proband confirming c.139-1G>A and c.724_725del, respectively, with the corresponding reference sequences shown for comparison. (**I**) Schematic of the PYCR1 transcript and protein showing exon 3 skipping with predicted p.(Met48_Lys107del) and the frameshift p.(Leu242AlafsTer31). In (**F**), the filled black symbol indicates the affected individual, central dots indicate heterozygous carriers, the arrow identifies the proband, red markers correspond to the variant alleles shown in the legend, and asterisks identify individuals who underwent genetic testing. Electropherogram colors in (**G**,**H**) follow standard nucleotide color coding.

**Figure 2:**
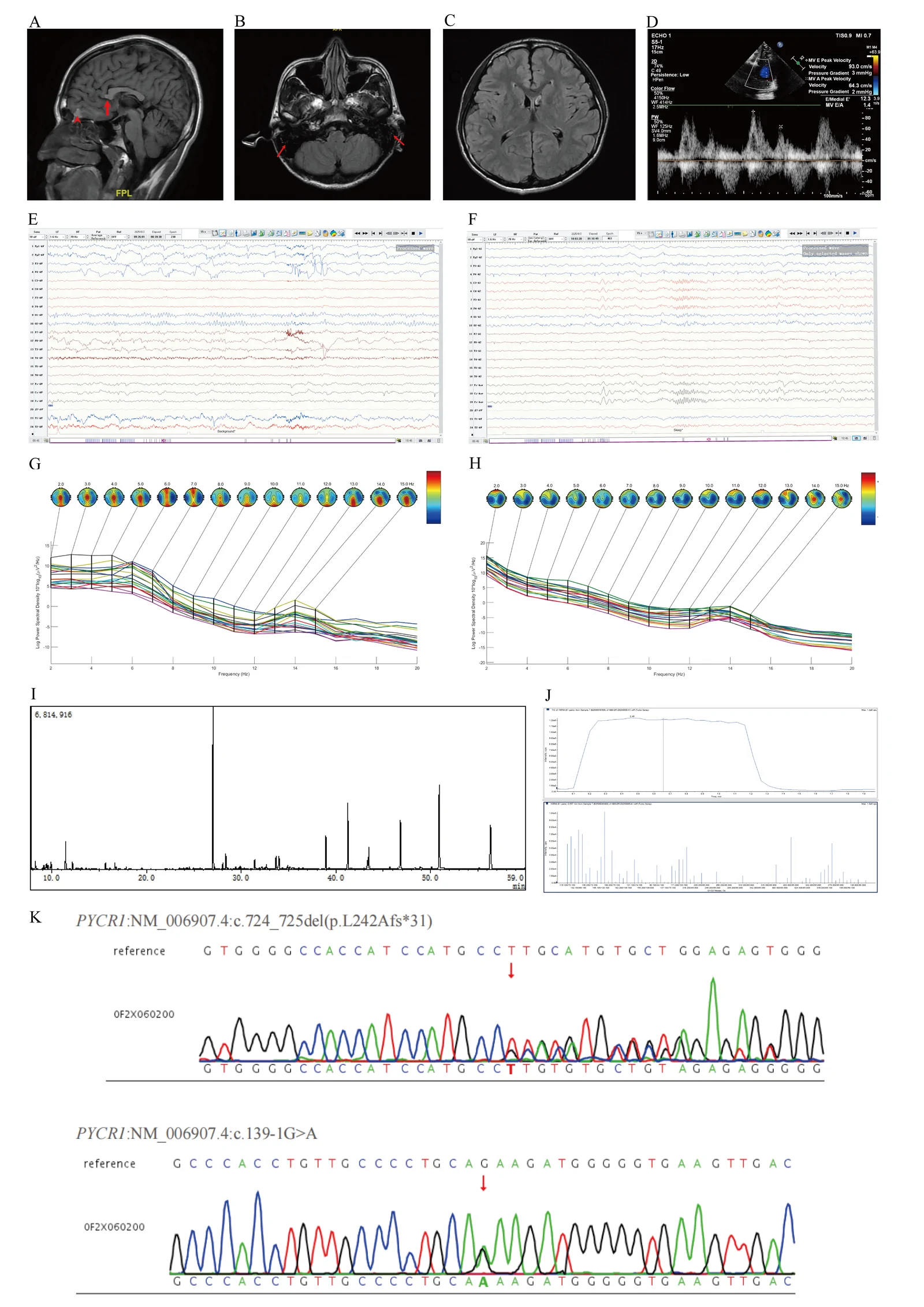
Ancillary investigations of the proband. (**A**–**C**) Brain MRI, including a mid-sagittal image demonstrating irregular morphology of the anterior corpus callosum. (**D**) Echocardiography. (**E**,**F**) EEG recordings obtained during awake and sleep states. (**G**,**H**) Exploratory power spectral density profiles of the proband and age-matched control, respectively. (**I**,**J**) Plasma and urine metabolic screening. Specifically, (**I**) urinary organic-acid screening by gas chromatography–mass spectrometry and (**J**) dried-blood-spot amino-acid and acylcarnitine screening by tandem mass spectrometry are shown. (**K**) Sanger sequencing traces of the two PYCR1 variants. Red arrows highlight the findings indicated in panels (**A**,**B**,**K**); FPL is a scanner-generated orientation marker. Colors in panels (**E**–**H**) are generated by the EEG acquisition and analysis software, and the color scales in (**G**,**H**) indicate relative spectral power. Chinese text in (**D**) consists of device-generated echocardiographic measurement labels.

The 16-year-old male proband presented with physical features consistent with PYCR1-related ARCL after molecular confirmation. Examination showed generalized joint hypermobility, thin lax skin with prominent superficial veins, pectus excavatum, genu varum, pes planus, increased plantar skin markings, and characteristic facial features including forehead wrinkling and prominent pinnae (Figure 1A–E). Motor developmental delay and reduced muscle bulk were also noted. Routine blood, hepatic, renal, thyroid, cardiac, and metabolic investigations were unremarkable. Brain MRI demonstrated irregular morphology of the anterior corpus callosum (Figure 2A). EEG recordings and exploratory PSD analysis are described below. The parents reported no consanguinity.

### 3.2. Identification of Novel Compound-Heterozygous PYCR1 Variants

Whole-exome sequencing identified two previously unreported compound-heterozygous PYCR1 variants based on NM_006907.4: the maternally inherited splice-acceptor variant c.139-1G>A and the paternally inherited frameshift variant c.724_725del, predicted to result in p.(Leu242AlafsTer31). Sanger sequencing confirmed familial segregation, establishing the two variants in trans. The maternal half-sister carried neither variant. No additional likely pathogenic variants or exome-derived CNVs explaining the phenotype were identified. Both variants were absent from the population databases examined, supporting PM2_Supporting. The genetic findings, segregation pattern, and variant positions are summarized in the composite genetic figure.

### 3.3. Endogenous RNA Analysis of the PYCR1 c.139-1G>A Splice Variant

**Figure 3:**
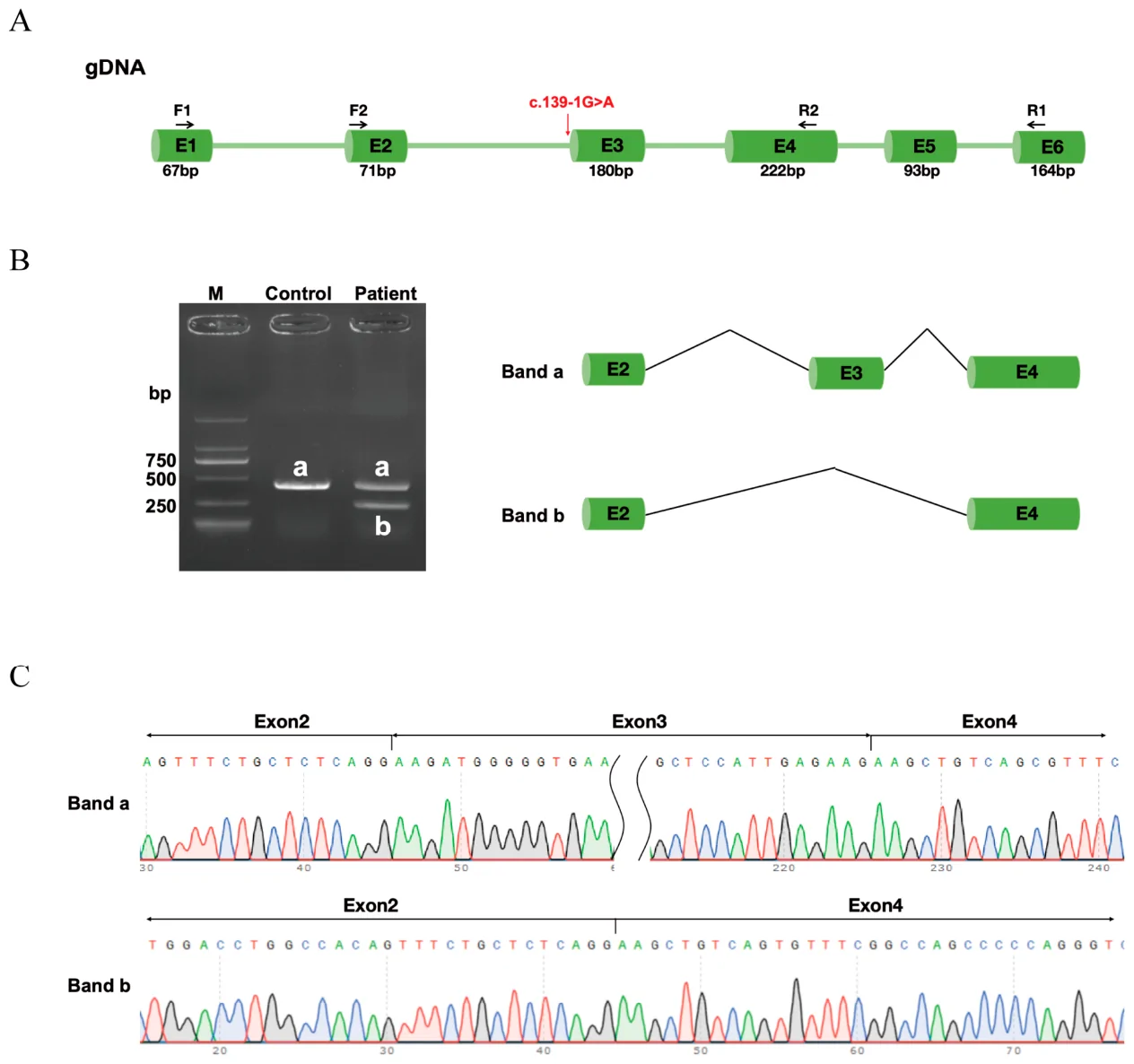
Endogenous RNA analysis of the PYCR1 c.139-1G>A splice variant. (**A**) Nested RT-PCR primer design, with c.139-1G>A indicated. (**B**) Agarose-gel analysis showing the 422-bp normally spliced product and the 242-bp aberrant product in the proband. (**C**) Sanger sequencing of the aberrant product confirming the exon 2–exon 4 junction. In (**A**), green boxes represent exons and the red arrow marks c.139-1G>A. In (**B**), bands a and b denote the normally spliced and exon 3-skipped products, respectively. Electropherogram colors follow standard nucleotide color coding. The assay was qualitative and was used to identify and sequence the splice products.

Qualitative nested RT-PCR of peripheral-blood RNA identified the expected 422-bp product in the unrelated healthy control and both the 422-bp product and a 242-bp product in the proband (Figure 3). Because the primers anneal within exons 2 and 4, the 422-bp amplicon contains the complete 180-bp exon 3 plus portions of the flanking exons; exon 3 skipping therefore reduces the product to 242 bp. Sanger sequencing of the shorter cloned product confirmed the exon 2-exon 4 junction, directly demonstrating an exon 3-skipped transcript associated with c.139-1G>A. The observed RNA consequence is r.139_318del and predicts the in-frame protein consequence p.(Met48_Lys107del), deleting 60 of 319 amino acids (~18.8%). The deleted segment lies within the N-terminal Rossmann-like NAD(P)H-binding region. Maternal RNA was not analyzed; therefore, allele-specific residual normal splicing from the maternal c.139-1G>A allele was not assessed. The endpoint nested RT-PCR assay was used for splice-product identification and sequence confirmation rather than quantitative transcript measurement.

### 3.4. Expression-Construct Analysis of the PYCR1 c.724_725del Variant

**Figure 4:**
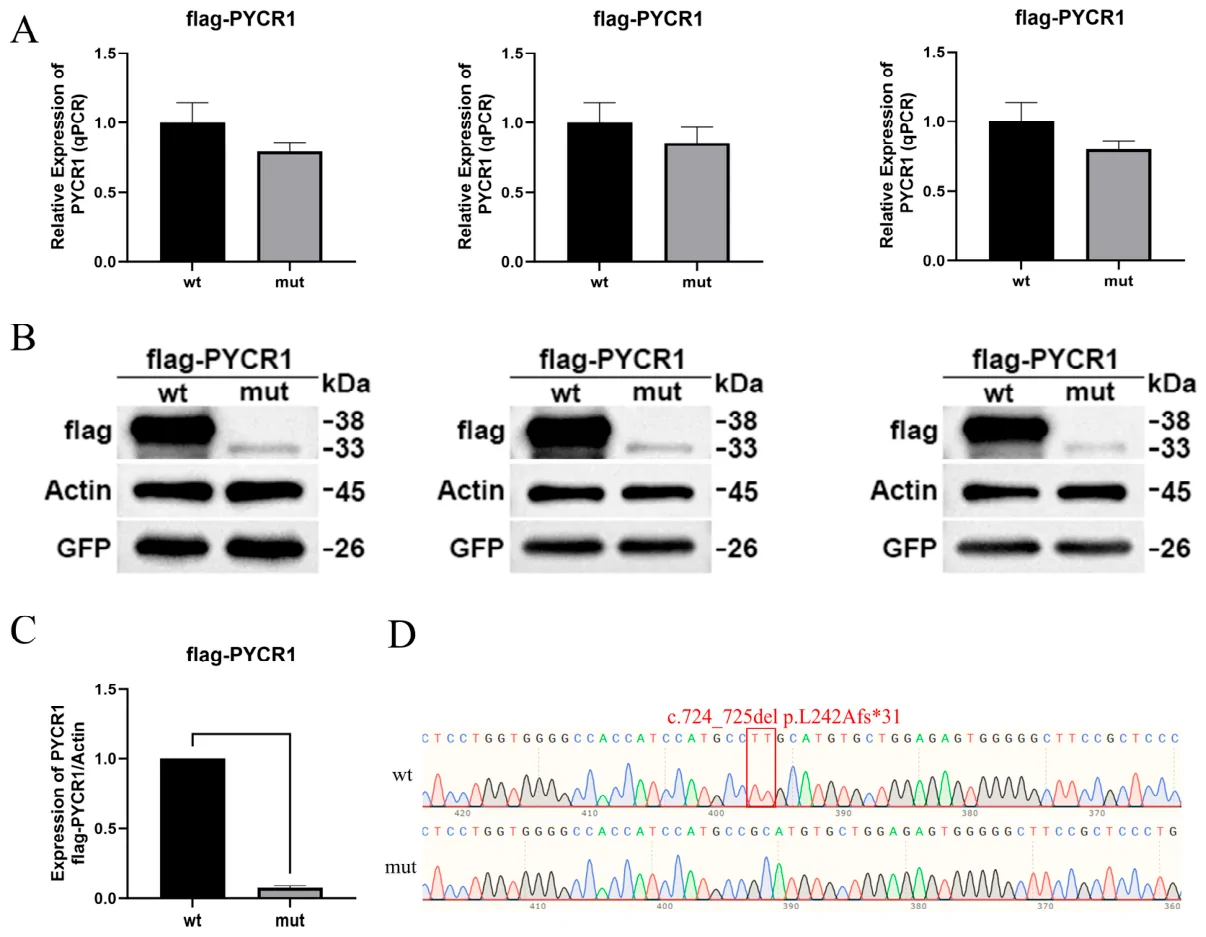
Expression-construct analysis of the PYCR1 c.724_725del variant. (**A**) Construct-derived PYCR1 transcript abundance in HEK293T cells transfected with WT or c.724_725del constructs; all values from three independent experiments are displayed. (**B**) Representative Western blot of N-terminal 3×FLAG-tagged WT and c.724_725del PYCR1. β-actin served as the loading control; GFP, expressed independently from the same plasmid, was used to monitor transfection. (**C**) Densitometric analysis of FLAG-PYCR1 abundance from three independent experiments. Band intensities were quantified using ImageJ and normalized to β-actin; the three independent experiments are summarized descriptively using the mean and observed range. (**D**) Sanger sequencing confirming the WT and c.724_725del expression constructs. Original uncropped Western blot images of FLAG-tagged PYCR1, GFP, β-actin, and GAPDH corresponding to Figure 4B are provided in Supplementary File S1.

WT and c.724_725del PYCR1 expression constructs were generated and verified by Sanger sequencing (Figure 4D). EGFP, expressed independently from the same plasmid, served as a transfection reporter. qPCR showed consistently lower construct-derived PYCR1 transcript abundance for c.724_725del than for WT across the three independent experiments (Figure 4A); this construct-associated transcript finding was not interpreted as evidence of endogenous nonsense-mediated decay. Western blotting with anti-FLAG antibody detected the WT and truncated PYCR1 proteins at approximately 38 and 33 kDa, respectively (Figure 4B). Across all three independent experiments, the c.724_725del construct showed lower steady-state abundance of the truncated PYCR1 protein than WT (Figure 4C), providing variant-specific functional evidence for an adverse effect on protein abundance in this heterologous expression system.

### 3.5. ACMG/AMP Classification of the PYCR1 Variants

The two PYCR1 variants were classified using the ACMG/AMP framework with relevant ClinGen criterion-specific recommendations; RNA and protein evidence were evaluated separately to avoid double counting.

The c.724_725del variant causes a frameshift beginning at Leu242 and is predicted to result in p.(Leu242AlafsTer31). The newly generated termination codon is located in the terminal exon of NM_006907.4; therefore, canonical exon-junction-complex-dependent nonsense-mediated decay is not expected. Nevertheless, the frameshift alters the native sequence from residue 242 onward and introduces premature termination, disrupting the C-terminal region. PVS1_Strong was applied under the ClinGen framework for an NMD-escaping truncating variant affecting a functionally relevant region. The variant was absent from the population databases examined (PM2_Supporting). The c.724_725del construct showed reduced steady-state abundance of the truncated PYCR1 protein relative to WT; given the scope of the heterologous expression assay, this evidence was applied as PS3_Supporting. The combined evidence supported a likely pathogenic classification.

The c.139-1G>A variant affects the canonical splice-acceptor site preceding exon 3. Endogenous RNA analysis directly demonstrated exon 3 skipping, yielding r.139_318del and predicting p.(Met48_Lys107del), a 60-amino-acid in-frame deletion (~18.8% of PYCR1) within the N-terminal Rossmann-like NAD(P)H-binding region. Because exon 3 is constitutively included in the MANE Select transcript and the observed splice alteration removes a substantial functionally relevant region while preserving the reading frame, PVS1_Strong(RNA), rather than PVS1_VeryStrong, was applied. The RNA result was incorporated into PVS1 and was not additionally counted as PS3. The variant was absent from the population databases examined (PM2_Supporting; Figure S2) and occurred in trans with the paternally inherited likely pathogenic c.724_725del variant (PM3). The combined evidence supported a likely pathogenic classification. Evidence codes are summarized in Table 2.

### 3.6. Neurodevelopmental Abnormalities in the Patient

Brain MRI demonstrated irregular morphology of the anterior corpus callosum (Figure 2A). EEG showed a posterior dominant rhythm with appropriate reactivity and modulation (Figure 2E,F). Exploratory PSD analysis provided a quantitative description of the patient’s spectral profile, showing bilateral symmetry across the 2–12 Hz range and relatively greater power at selected midline electrodes compared with the age-matched control (Figure 2G,H) [30]. These patient-level electrophysiological features are presented as descriptive, hypothesis-generating observations rather than a disease-specific EEG signature. On the Chinese WAIS-IV, the patient showed a markedly uneven cognitive profile, with a Verbal Comprehension Index (VCI) of 52 and a Perceptual Reasoning Index (PRI) of 94, a 42-point inter-index discrepancy; FSIQ was 75. The domain-level pattern indicates pronounced verbal-comprehension weakness with relatively preserved nonverbal perceptual reasoning, and the verbal findings were interpreted in the context of the patient’s developmental and language characteristics.

### 3.7. Spatiotemporal Expression Profile of PYCR1 in Development and Brain

**Figure 5:**
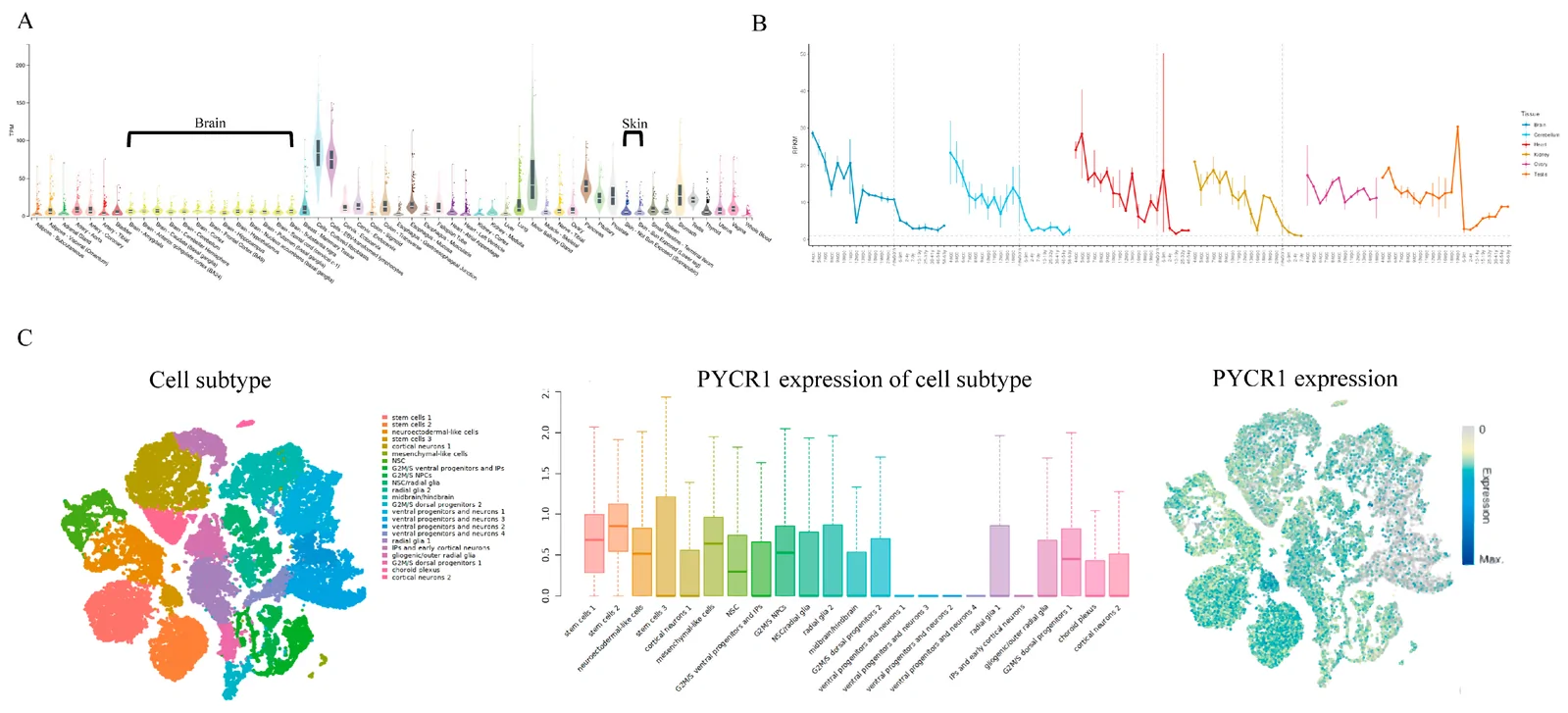

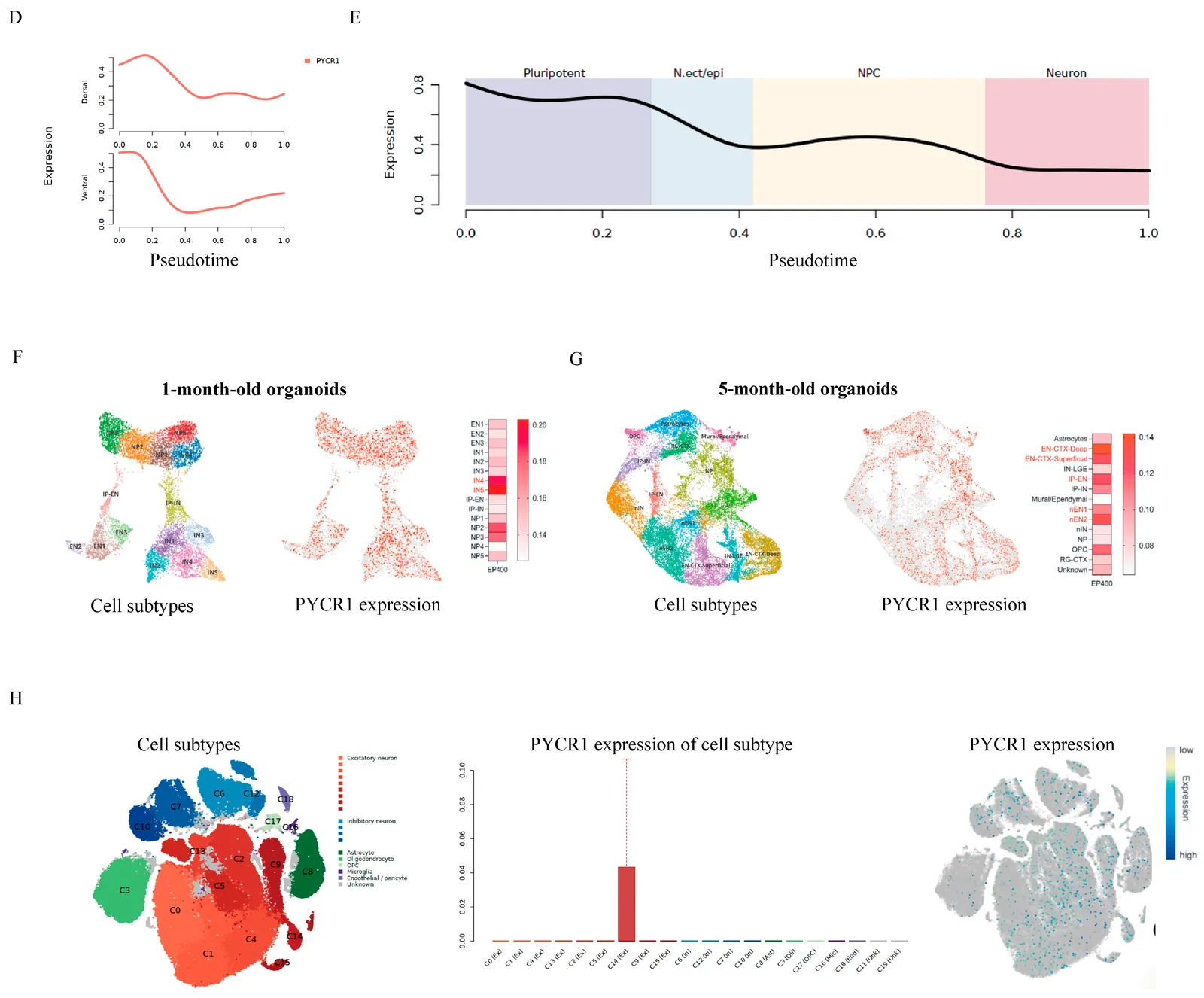
Spatiotemporal expression context of PYCR1 across public transcriptomic and single-cell datasets. (**A**) Adult human tissue expression from the GTEx Portal. (**B**) Developmental organ expression from the Evo-devo mammalian organs dataset (human dataset: ArrayExpress E-MTAB-6814). (**C**) PYCR1 expression across cell populations during human cerebral-organoid development using processed scRNA-seq data from Kanton et al. available through ScApeX (E-MTAB-7552). (**D**) PYCR1 expression along the source-study developmental trajectory toward the telencephalic lineage. (**E**) PYCR1-associated chromatin-accessibility dynamics using human organoid scATAC-seq data (E-MTAB-8089). (**F**,**G**) PYCR1 expression in 1- and 5-month single-neural-rosette-derived cortical organoids using the Wang et al. dataset available through the Human Organoid Single-Cell Browser (GEO GSE118697). (**H**) Adult human brain cell populations using the Kanton et al. snRNA-seq dataset (E-MTAB-8230). Processed expression values, cell labels, and trajectory information were based on the corresponding source resources and original studies. Colors are panel-specific and correspond to the cell-type or expression-scale legends shown within panels (**C**,**F**–**H**); colors are not intended to match across datasets.

Integration of public transcriptomic and single-cell multi-omics datasets revealed developmentally and cell-type-variable PYCR1 expression (Figure 5). PYCR1 was broadly expressed across adult tissues and showed dynamic expression across developmental organs. In cerebral-organoid datasets, PYCR1 showed relative enrichment in early neural progenitor populations and changes along neural differentiation trajectories; complementary scATAC-seq data showed developmental changes at the PYCR1 locus. Single-neural-rosette organoid datasets showed stage-dependent differences in expression across progenitor and neuronal populations, while adult-brain snRNA-seq showed generally lower expression with relative enrichment in selected neuronal subpopulations. These observations provide spatiotemporal biological context and are interpreted as associative rather than mechanistic.

## 4. Discussion

We identified two previously unreported PYCR1 variants in trans and obtained complementary variant-specific functional evidence at the RNA and protein levels. Endogenous RNA analysis demonstrated that c.139-1G>A alters PYCR1 splicing, producing an exon 3-skipped transcript, whereas expression-construct experiments showed consistently reduced steady-state abundance of the truncated p.(Leu242AlafsTer31) protein. Together with familial segregation and population evidence, these findings strengthen the molecular interpretation of both variants and extend the molecular spectrum of PYCR1-related disease.

The RNA and expression-construct assays address distinct molecular consequences. Exon 3 skipping is predicted to produce p.(Met48_Lys107del), deleting 60 amino acids (~18.8% of the protein) from the N-terminal Rossmann-like NAD(P)H-binding region, providing structural context for the experimentally demonstrated splice alteration. For c.724_725del, the frameshift is predicted to escape canonical NMD because the new termination codon lies in the terminal exon, but it alters the native sequence from Leu242 onward and terminates prematurely. The observed reduction in steady-state abundance of the truncated protein provides complementary variant-specific functional evidence. The construct-derived transcript result is interpreted separately and not as evidence of endogenous NMD; catalytic activity remains to be evaluated in a validated enzymatic system.

A review of previously reported molecularly confirmed PYCR1-related cases demonstrates substantial allelic and phenotypic heterogeneity, including homozygous and compound-heterozygous genotypes and multiple variant classes. Previous genotype–phenotype studies have suggested regional trends, with variants affecting exons 4-6 associated, on average, with greater clinical severity, although substantial phenotypic overlap occurs across variant locations and classes [15]. The present patient carries an exon 3 splice-altering variant and an exon 6 frameshift variant with distinct molecular consequences. These findings are compatible with the reported regional trend while supporting a spectrum-based rather than deterministic interpretation of genotype–phenotype relationships.

The patient’s cutaneous, facial, joint, musculoskeletal, and developmental manifestations are broadly consistent with the reported phenotypic spectrum of PYCR1-related ARCL. Detailed neurodevelopmental assessment additionally revealed a markedly uneven cognitive profile, with pronounced verbal-comprehension weakness and relatively preserved nonverbal perceptual reasoning. The irregular morphology of the anterior corpus callosum represents an additional neuroimaging observation in this molecularly confirmed case and is presented descriptively rather than as a disease-specific imaging marker.

Exploratory PSD analysis adds quantitative electrophysiological characterization to the clinical, genetic, and neuroimaging findings. The observed spectral features are patient-level, hypothesis-generating observations; they are not interpreted as a PYCR1-specific EEG signature or as demonstrating an anatomical correspondence with corpus callosum morphology. Evaluation in additional molecularly characterized individuals will be useful for determining their reproducibility and phenotypic relevance.

Integration of complementary public transcriptomic and single-cell multi-omics resources provided additional spatiotemporal context for the potential neurodevelopmental relevance of PYCR1. Across cerebral-organoid datasets, PYCR1 showed relative enrichment in early neural progenitor populations and dynamic changes along neural differentiation trajectories, with complementary developmental changes in chromatin accessibility at the PYCR1 locus. Stage-dependent differences were also observed across neuronal populations, while adult-brain expression was generally lower. These convergent observations provide biological context and generate testable hypotheses regarding neural differentiation; they are not interpreted as evidence that PYCR1 is a primary regulator of neurogenesis or that the identified variants directly alter excitatory/inhibitory neuronal balance.

Routine blood and urine metabolic screening was unremarkable, consistent with prior observations that PYCR1-related disease may not be detected by standard metabolic screening alone. The molecular diagnosis therefore relied on the integration of clinical findings, genomic testing, segregation analysis, and variant-specific functional evidence.

The principal strengths of this study are the identification of two previously unreported variants in trans, direct demonstration of an aberrant endogenous RNA splice product for c.139-1G>A, and complementary protein-abundance evidence for c.724_725del, together with detailed clinical and neurodevelopmental phenotyping. The RNA assay was qualitative, and maternal RNA was not analyzed, while the heterologous expression assay assessed steady-state abundance rather than catalytic activity. These boundaries define the scope of the functional evidence and provide clear priorities for future allele-specific RNA and enzymatic studies.

Overall, the combined genetic, RNA, protein, clinical, and public-data observations extend the reported PYCR1 allelic and phenotypic spectrum. Continued accumulation of molecularly characterized cases with standardized phenotyping and variant-specific functional data will help refine genotype–phenotype relationships and the neurodevelopmental context of PYCR1-related disease.

## 5. Conclusions

This study identifies two previously unreported compound-heterozygous PYCR1 variants in trans in a patient with PYCR1-related ARCL. Endogenous RNA analysis directly demonstrated exon 3 skipping associated with c.139-1G>A, while expression-construct experiments showed consistently reduced steady-state abundance of the truncated protein encoded by c.724_725del. Integration of segregation, population, and variant-specific functional evidence supported likely pathogenic classification of both variants. Detailed clinical and neurodevelopmental characterization, together with complementary public-data analyses, extends the molecular and phenotypic spectrum of PYCR1-related disease and provides additional evidence relevant to molecular diagnosis, variant interpretation, and genetic counseling.

## Figures and Tables

**Table 1:** Primers used for genomic variant confirmation, endogenous RNA analysis, vector construction, and qPCR.

Application	Primer Name	Primer Sequence (5′→3′)	Purpose/Notes
Genomic PCR/Sanger confirmation: c.139-1G>A	hg38-PYCR1-81935517-F	CATTCTCTGTGCCATTCTACCTG	PCR forward primer; 193-bp amplicon
Genomic PCR/Sanger confirmation: c.139-1G>A	hg38-PYCR1-81935517-R	CCAGGATGAAGGGGATGATG	PCR reverse primer and reverse-direction cycle-sequencing primer; 193-bp amplicon
Genomic PCR/Sanger confirmation: c.724_725del	hg38-PYCR1-81934398-F	GGGCTGCCAAGATGCTGC	PCR forward primer and forward-direction cycle-sequencing primer; 258-bp amplicon
Genomic PCR/Sanger confirmation: c.724_725del	hg38-PYCR1-81934398-R	TATCTGCTGAGTGCGTGAATGA	PCR reverse primer; 258-bp amplicon
Endogenous RNA analysis	1-F	TGGCTTTTGCCCTGGCCAAG	Nested RT-PCR
Endogenous RNA analysis	1-R	GGGTGCTGTTCTGAGTGCAG	Nested RT-PCR
Endogenous RNA analysis	2-F	CGTCTTGGCTGCCCACAAGA	Nested RT-PCR
Endogenous RNA analysis	2-R	CTTCCACCTCCGTGCAGAAG	Nested RT-PCR
Vector construction	pCMV-3XFlag-Neo(EGFP)-PYCR1-NotI-F	gcttgcggccgccATGAGCGTGGGCTTCAT	Amplification and cloning of the wild-type PYCR1 coding sequence
Vector construction	pCMV-3XFlag-Neo(EGFP)-PYCR1-EcoRI-R	cgatgaattcTCAATCCTTGCCCGCTGGGG	Amplification and cloning of the wild-type PYCR1 coding sequence
Vector construction	PYCR1-mut-F	GCCACCATCCATGCCGCATGTGCTGGAGAG	Generation of the c.724_725del construct by overlap PCR
Vector construction	PYCR1-mut-R	CTCTCCAGCACATGCGGCATGGATGGTGGC	Generation of the c.724_725del construct by overlap PCR
qPCR	PYCR1-qPCR-F	AGCCACACATCATCCCCTTC	Detection of construct-derived PYCR1 transcripts
qPCR	PYCR1-qPCR-R	CGACTGGAGTGTTGGTCATG	Detection of construct-derived PYCR1 transcripts
qPCR	ACTB-qPCR-F	TGACGTGGACATCCGCAAAG	Reference-gene amplification
qPCR	ACTB-qPCR-R	CTGGAAGGTGGACAGCGAGG	Reference-gene amplification

Note: All primer sequences are shown in the 5′→3′ orientation. Both forward and reverse primers were used for genomic PCR amplification. The 193-bp amplicon encompassing c.139-1G>A was sequenced in the reverse direction using hg38-PYCR1-81935517-R, whereas the 258-bp amplicon encompassing c.724_725del was sequenced in the forward direction using hg38-PYCR1-81934398-F. The corresponding opposite-direction primers were used for PCR amplification but not for cycle sequencing. Lowercase nucleotides in the vector-construction primers indicate added cloning overhangs and restriction-enzyme recognition sequences. ACTB was used as the reference gene for qPCR.

**Table 2:** ACMG/AMP classification of the two *PYCR1* variants.

Variant	Molecular Consequence	Evidence and Rationale	Classification
NM_006907.4:c.724_725del (p.(Leu242AlafsTer31))	Frameshift beginning at Leu242; predicted p.(Leu242AlafsTer31); terminal-exon premature termination, with canonical EJC-dependent NMD not expected	PVS1_Strong: NMD-escaping truncating variant disrupting the native C-terminal sequence; PM2_Supporting: absent from population databases examined; PS3_Supporting: reduced steady-state abundance of the truncated protein in the heterologous expression system	Likely pathogenic
NM_006907.4:c.139-1G>A	Endogenous RNA: r.139_318del (exon 3 skipping); predicted p.(Met48_Lys107del), deleting 60 amino acids in frame within the N-terminal Rossmann-like NAD(P)H-binding region	PVS1_Strong(RNA): exon 3 skipping directly demonstrated in endogenous RNA and substantial in-frame deletion of a functionally relevant region; PM2_Supporting: absent from population databases examined; PM3: confirmed in trans with c.724_725del	Likely pathogenic

Abbreviations: ACMG/AMP, American College of Medical Genetics and Genomics/Association for Molecular Pathology; LP, likely pathogenic; RNA, ribonucleic acid. The RNA splicing result for c.139-1G>A was incorporated into PVS1_Strong(RNA) and was not additionally counted as PS3. PS3_Supporting for c.724_725del was based on reduced protein abundance rather than a direct measurement of PYCR1 enzymatic activity.

## Data Availability

The data supporting the findings of this study are available from the corresponding authors on reasonable request. Because this study includes potentially identifiable clinical information from a single patient, deidentified data will be shared only to the extent permitted by institutional ethics approval and patient privacy requirements.

## Supplementary Materials

The following supporting information can be downloaded at https://www.mdpi.com/article/10.3390/genes17091129/s1, Figure S1: Representative EGFP fluorescence images used to monitor transfection of the pCMV-3XFlag-Neo(EGFP)-PYCR1 WT and c.724_725del constructs. Figure S2: In silico prediction and population-database query results for the identified PYCR1 variants. Supplementary File S1: Original uncropped Western blot images of FLAG-tagged PYCR1, GFP, β-actin, and GAPDH corresponding to Figure 4B.

## Abbreviations

1000G	1000 Genomes Project
ACMG/AMP	American College of Medical Genetics and Genomics/Association for Molecular Pathology
ARCL	Autosomal recessive cutis laxa
CNV	Copy-number variation
EEG	Electroencephalography
ExAC	Exome Aggregation Consortium
FSIQ	Full-Scale Intelligence Quotient
gnomAD	Genome Aggregation Database
GTEx	Genotype-Tissue Expression
LP	Likely pathogenic
NMD	Nonsense-mediated mRNA decay
NPCs	Neural progenitor cells
NSC	Neural stem cell
P5C	Pyrroline-5-carboxylate (or Δ^1^-pyrroline-5-carboxylate)
PRI	Perceptual Reasoning Index
PSD	Power spectral density
PYCR1	Pyrroline-5-carboxylate reductase 1
scATAC-seq	Single-cell assay for transposase-accessible chromatin sequencing
scRNA-seq	Single-cell RNA sequencing
snRNA-seq	Single-nucleus RNA sequencing
VCI	Verbal Comprehension Index
VUS	Variant of uncertain significance
WAIS-IV	Wechsler Adult Intelligence Scale, Fourth Edition
WES	Whole-exome sequencing
