# Supplementary material for: Novel Compound-Heterozygous Variants in PYCR1 Expand the Variant Spectrum of Autosomal Recessive Cutis Laxa
Source: Genes (Basel). 2026 Sep 16;17(9):1129. doi: 10.3390/genes17091129 (PMC13606760; doi:10.3390/genes17091129)
Supplement: Supplementary file 1 [file genes-17-01129-s001.zip › Supplementary File S1.pdf]

Supplementary File S1

Original uncropped Western blot images of FLAG-tagged PYCR1, GFP, β-actin, and GAPDH corresponding to Figure 4B

Images are displayed without cropping or tonal adjustment. Original TIFF files were converted losslessly to PNG solely for embedding in this Word document. Images are grouped according to the replicate identifiers in the supplied source filenames, which are retained below each image.

Replicate 1

β-actin

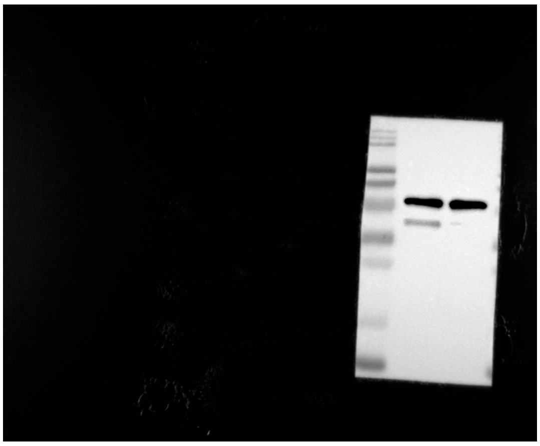

Original file: Figure4B_Rep1_ original-ACTIN(2).tif

FLAG

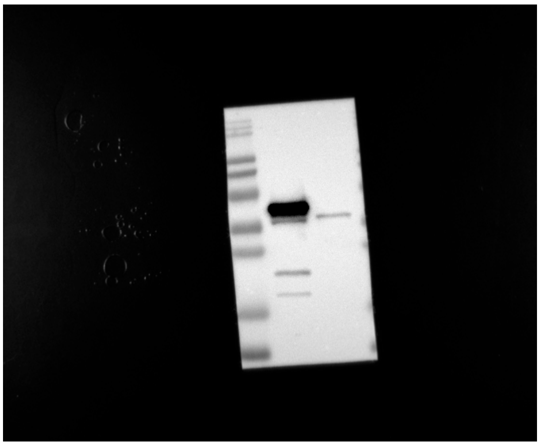

Original file: Figure4B_Rep1_ original-FLAG(2).tif

GAPDH

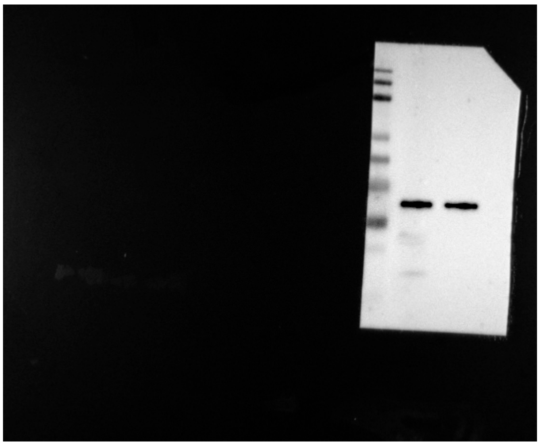

Original file: Figure4B_Rep1_ original-GAPDH(2).tif

GFP — source image 1

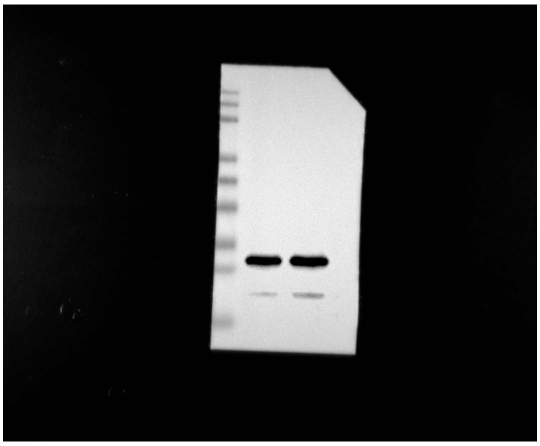

Original file: Figure4B_Rep1_ original-GFP(4).tif

GFP — source image 2

Replicate 2

β-actin

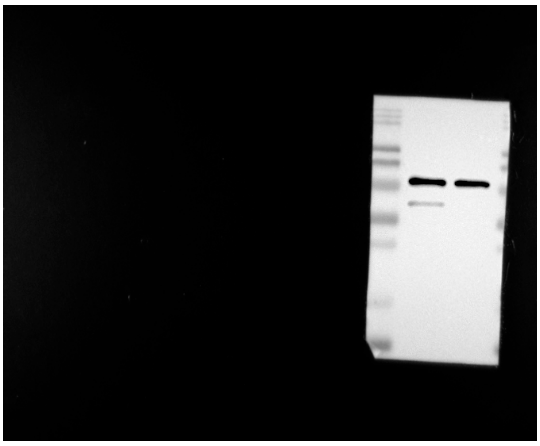

Original file: Figure4B_Rep2_ original-ACTIN(2).tif

FLAG

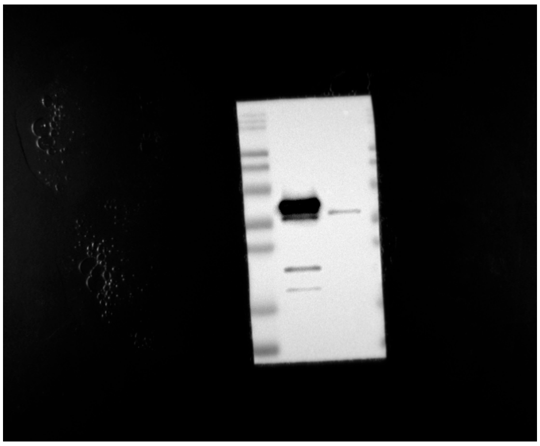

Original file: Figure4B_Rep2_ original-FLAG(2).tif

GAPDH

β-actin

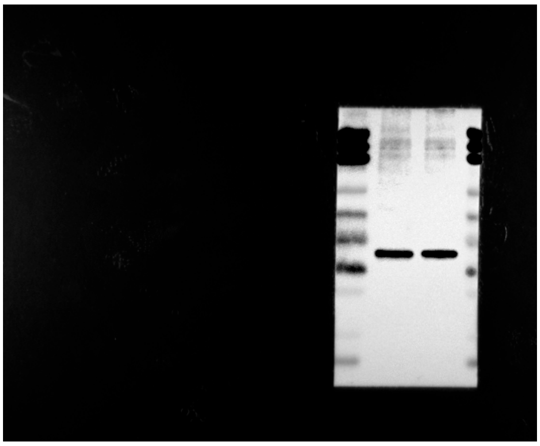

Original file: Figure4B_Rep2_ original-GAPDH(2).tif

FLAG

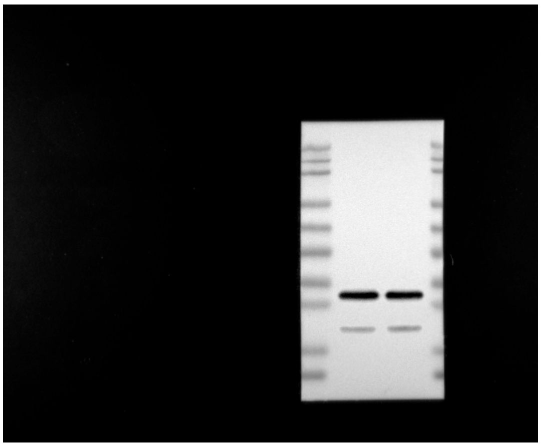

Original file: Figure4B_Rep2_ original-GFP(4).tif

Replicate 3

β-actin

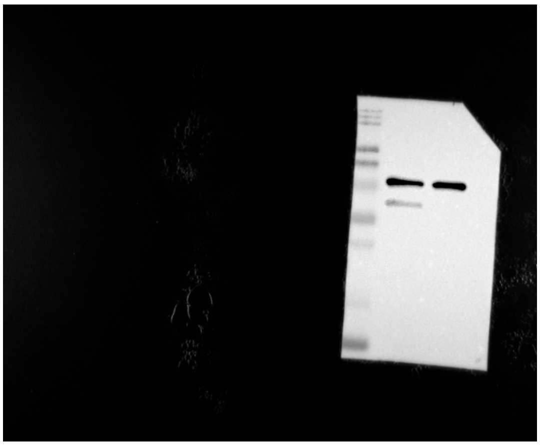

Original file: Figure4B_Rep3_ original-ACTIN(2).tif

FLAG

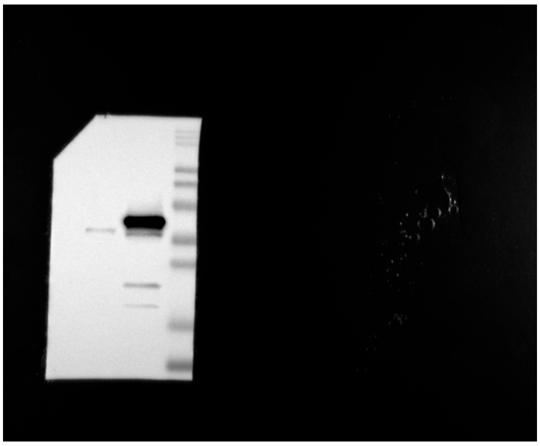

Original file: Figure4B_Rep3_ original-FLAG(2).tif

GAPDH

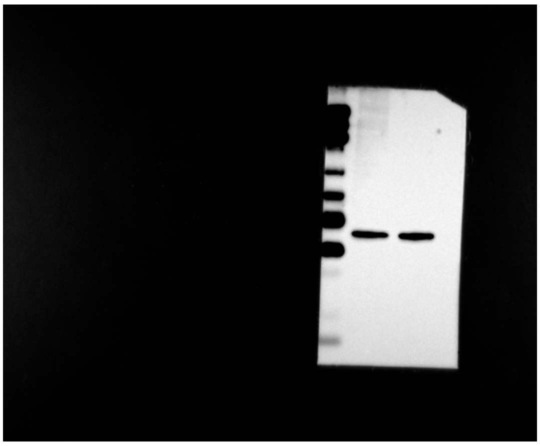

Original file: Figure4B_Rep3_ original-GAPDH(2).tif

GFP

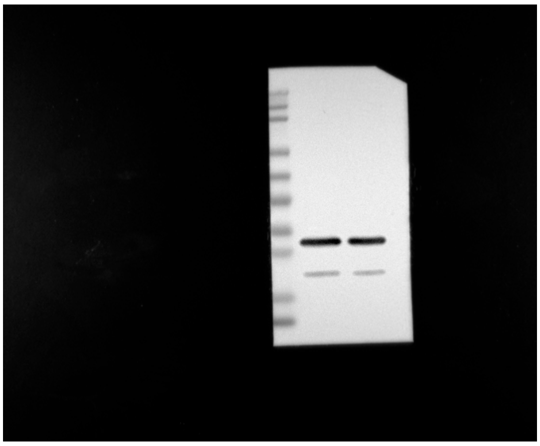

Original file: Figure4B_Rep3_ original-GFP(2).tif
